# Omega-3 fatty acids, polymorphisms and lipid related cardiovascular disease risk factors in the Inuit population

**DOI:** 10.1186/1743-7075-10-26

**Published:** 2013-03-12

**Authors:** Iwona Rudkowska, Catherine Ouellette, Eric Dewailly, Robert A Hegele, Véronique Boiteau, Ariane Dubé-Linteau, Belkacem Abdous, Françoise Proust, Yves Giguère, Pierre Julien, Marie-Ludivine Château-Degat, Marie-Claude Vohl

**Affiliations:** 1 Institute of Nutrition and Functional Foods (INAF), Laval University, 2440, boulevard Hochelaga, Québec, QC, Canada; 2 Laboratory of Endocrinology and Nephrology, Laval University Hospital Research Center, Quebec, QC, Canada; 3 Laboratory of Population and Environmental Health, Laval University Hospital Research Center, Quebec, QC, Canada; 4 Robarts Research Institute, London, ON, Canada

**Keywords:** Nutrigenetics, n-3 PUFAs, Plasma lipids, Gene-nutrient interactions

## Abstract

**Background:**

Tissue concentrations of fatty acids (FAs) and genetic variations are well-known factors which affect the cardiovascular disease (CVD) risk. The objective was to examine whether the genetic variability of 20 candidate genes and red blood cells (RBCs) percentage of total *n*-3 polyunsaturated fatty acids (PUFA), a biomarker of dietary *n*-3 PUFA intake, modulate lipid related CVD risk factors in the Inuit population.

**Methods:**

Data from the Qanuippitaa Nunavik Health Survey (n = 553) were analysed via multivariate regression models with 40 known polymorphisms, RBCs percentage of *n*-3 PUFA, and the interaction term to take into account the effect on plasma lipid and apolipoporotein levels.

**Results:**

Individuals being heterozygotes for *CETP* C-4502T (rs183130) or G-971A (rs4783961) together with higher *n*-3 PUFA had lower triacylglycerol (TG) concentrations compared to homozygotes for the minor allele. Further, effects of a stronger beneficial association between *n*-3 PUFA in RBCs and plasma lipid parameters- including lower total cholesterol (TC), lower low-density lipoprotein cholesterol (LDL-C) or higher high-density lipoprotein cholesterol (HDL-C) concentrations- were associated with *AGT* M235T (rs699) TT genotype, *CETP* G-971A (rs4783961) AG genotype, T allele carriers of *CETP* C-4502T (rs183130), and T allele carriers of *CETP* Ile405Val (rs5882). In contrast, higher *n*-3 PUFA in RBCs were associated with adverse lipid profiles- including increased LDL-C, increased apolipoprotein B100 or decreased HDL-C concentrations- in G allele carriers of the *APOA5* -3 A/G (rs651821), C allele carriers of *APOA5* T-1131C (rs662799), G carriers of *APOC3* SstI (rs5128) and G carriers of *APOA4* Asn147Ser (rs5104).

**Conclusion:**

Overall, these results suggest that percentage of total *n*-3 PUFA of RBCs are associated with lipids related CVD risk factors conferred by genetic variations in the Inuit population.

## Background

The lifestyle of the Inuit is rapidly changing towards an increased cardiovascular (CVD) risk factor profile [1]. The CVD risk factors include non-modifiable risk factors such as age, male sex, family history, and modifiable risk factors for example smoking, hypertension, diabetes, and hyperlipidemia. Specifically, improving plasma lipids profile– including: total cholesterol (TC), triacylglycerol (TG), low-density-lipoprotein cholesterol (LDL-C), apolipoprotein (apo) B concentrations, apo A1 concentrations and high-density-lipoprotein cholesterol (HDL-C)- is associated with a decreased CVD risk [2]. Studies have shown that both environmental and genetic factors may play a role in determining susceptibility to dyslipidemia.

The consumption of fish and marine mammals rich in *n*-3 PUFAs represents an important dietary component for CVD risk prevention in the Inuit population [3]. A high intake of *n*-3 polyunsaturated fatty acids (*n*-3 PUFAs), including eicosapentaenoic acid (EPA) (20:5(n-3)) and docosahexaenoic acid (DHA) (22:6(n-3)), has been shown to exert favorable effects on plasma lipid profiles [4]. Recently, a study confirmed that higher percentages of *n*-3 PUFA content in red blood cells (RBCs) were indicative of higher *n*-3 PUFA intake in the Inuit population [5]. Nonetheless, an increased risk of lipid related CVD is currently occurring despite the substantial proportion of the population maintaining traditional lifestyles including high intakes of *n*-3 PUFAs [6].

The Inuit populations also carry a unique distinct genetic background [7]. This genetic makeup has been shown in some studies to be protective; yet, others have reported potentially detrimental effects for CVD susceptibility [1,8]. These genetic variations may render an individual more or less responsive to changes in *n*-3 PUFA intake. Thus, gene-nutrient interactions may provide important insights into the inter-individual variability observed in plasma lipid levels and thus on the risk of CVD. Consequently, the main objective of this study was to examine whether the genetic variability of 20 candidate genes in lipid metabolism and percentage of total *n*-3 PUFA in RBCs - a biomarker of dietary *n*-3 PUFA intake, are associated with CVD risk factors including TG, TC, LDL-C, HDL-C, ratio TC/HDL-C and apoB100 concentrations.

## Methods

### Subjects

The data were collected in the framework of the Nunavik Inuit Health Survey “Qanuippitaa? How are we?” conducted among the Inuit of Nunavik in 2004. The survey covered all 14 Nunavik communities [6]. In the winter of 2009, participants from the Qanuippitaa Nunavik Health Survey in 2004 were revisited for the additional genetic component. Participants signed a consent form before data collection. Sampling strategies for the present population-based study have been described previously in detail [9]. All consent and assent forms were approved by the Ethics Committee on Research of Laval University and the Québec Public Health Ethics review board.

### Anthropometric measurements

Height was measured using a rigid square and a measuring tape in a standardized standing position, with the participant's back against the wall looking straight ahead, and arms hanging down freely. Body weight was measured with a scale (Tanita TBF-300, Manufacturer, Arlington Heights, IL). Body mass index (BMI) was calculated as weight per meter squared (kg/m^2^), and internationally recommended cutoffs were used [10].

### Plasma blood sampling

Blood samples were collected from an antecubital vein into vacutainer tubes containing EDTA. Plasma was separated by centrifugation (2500 x g for 10 min at 4°C) and samples were portioned and frozen for subsequent measurements. Plasma TC and TG concentrations were determined by enzymatic methods using a Hitachi 917 autoanalyser and reagents from Roche Diagnostics (Canada) [11]. The HDL-C fraction was obtained directly by precipitation with dextran sulfate-Mg2+ procedure [11]. The LDL-C was calculated with the Friedewald formula [12]. ApoB concentrations were measured in plasma by the rocket immunoelectrophoretic method of Laurell [13]. Lipid analyses were performed at the *Centre de Recherche sur les Maladies Lipidiques* (Centre de Recherche du CHUQ, Quebec, Qc, Canada).

### Food frequency questionnaire (FFQ)

A food-frequency questionnaire (FFQ) [14] was administered to the participants. A specifically designed 69-item FFQ was used to assess average food and beverage intakes during the previous year, as described in more details elsewhere [15]. Analysis of the FFQ data provided estimates of consumption frequency and the usual intake in grams of country foods on a daily, weekly, monthly, seasonal or an annual basis. Daily food intakes were calculated on an annual basis by multiplying food consumption frequency by intake in grams for each food.

### Fatty acid determination

Fatty acid (FA) composition of erythrocyte membranes was determined by gas chromatographic analysis. Membranes of lysed erythrocytes were isolated by centrifugation (21,000g, 15 minutes) and washed twice with 0.9% NaCl solution. Cell membranes were resuspended in 200 μL of the NaCl solution and were spiked with phosphatidylcholine C:15 (Avanti Polar Lipids, Alabaster, AL), used as internal standard. Lipids were extracted using a mixture of chloroform-methanol (2:1 v/v) according to a modified Folch method [16]. FA profiles were obtained after methylation in methanol/benzene 4:1 (v/v) [17] and capillary gas chromatography using a temperature gradient on a HP5890 gas chromatograph (Hewlett Packard, Toronto, Canada) equipped with a HP-88 capillary column (100m x 0.25 mm i.d. x 0.20μm film thickness; Agilent Technologies, Palo Atto, CA) coupled with a flame ionization detector (FID). Helium was used as carrier gas (split ratio 1:80). FA were identified according to their retention time, using the following standard mixtures as a basis for comparison: the FAME 37 mix (Supelco Inc., Bellefonte, PA) and the GLC-411 fatty acid mix (NuChek Prep Inc, Elysian, MN), as well as the following methylated fatty acids C22:5 n-6 (Larodan AB, Malmö, Sweden) and C22:5 n-3 (Supelco Inc., Bellefonte, PA). Erythrocyte FA profiles were expressed as the relative percentage areas of total FAs. Total percentage of *n*-3 PUFAs in RBCs was calculated from the sum of C18:3 (n-3), C18:4 (n-3), C20:4 (n-3), C20:5 (n-3) (EPA), C22:3 (n-3), C22:5 (n-3) (docosapentaenoic acid (DPA)), and C22:6 (n-3) (DHA).

### DNA extraction and genotyping

DNA was extracted from 100 μl of buffy coat using the QIAamp 96 DNA Blood Kit (QIAGEN Inc., Valencia, CA, USA). Quant-iT PicoGreen® dsDNA Assay Kit (Invitrogen) was used to quantify DNA. DNA was analysed with TaqMan® Pre-Designed SNP Genotyping Assays according to the manufacturer's instructions at the McGill University/Génome Québec Innovation Center (Montreal, Canada).

From a literature review, 40 polymorphisms in 20 genes relating to lipid metabolism were identified (Table 1) namely: *Angiotensin I converting enzyme (ACE)* I/D alleles (rs4343), *Angiotensinogen (AGT)* M235T (rs699), T174M (rs4762), A-20C (rs5050), A-6G (rs5051); *Apolipoprotein A-I (APOA1)* G-75A (rs670), 84 T/C (rs5070); *Apolipoprotein A-IV (APOA4)* Asn147Ser (rs5104)*; Apolipoprotein A-V (APOA5)* T-1131C (rs662799), -3 A/G (rs651821), S19W (rs3135506), Gly185Cys (rs2075291); *Apolipoprotein B (APOB)* XbaI (rs693); *Apolipoprotein C-III (APOC3)* SstI (rs5128), T-455C (rs2854116); *Apolipoprotein E (APOE)* Cys112Arg (rs429358), Arg158Cys (rs7412), G-219T (rs405509); *ATP-binding cassette, sub-family A (ABC1), member 1 (ABC1A)* Arg219Lys (rs2230806)*; Cholesteryl ester transfer protein (CETP)* TaqIB (rs708272), C-629A (rs1800775), C-4502T (rs183130), G-971A (rs4783961), Ile405Val (rs5882); *Cytochrome P450, family 1, subfamily A, polypeptide 1 (CYP1A1)* Msp1 (rs4646903), A4889G (rs1048943); *Fat mass and obesity associated (FTO)* (rs9939609)*; Glucokinase (hexokinase 4) regulator (GCKR)* (rs780094)*; Insulin induced gene 2 (INSIG2)* (rs7566605)*; Lipoprotein lipase (LPL)* HindIII (rs320), Ser447Ter (rs328); *Hepatic lipase (HL or LIPC)* C-514T (rs1800588), G-250A (rs2070895); *Methylenetetrahydrofolate reductase (NAD(P)H) (MTHFR)* C677T (rs1801133)*; Paraoxonase 1 (PON1)* L55M (rs854560), Gln192Arg (rs662), C-107T (rs705379); *Peroxisome proliferator-activated receptor gamma 2 (PPARG2)* Pro12Ala (rs1801282), 681 C/G (rs10865710); *Transcription factor 7-like 2 (TCF7L2)* C47833T (rs7903146).

**Table 1:** Genes, polymorphisms and the main function their protein

**Genes (Official symbol)**	**Polymorphisms**	**Main function of their protein**
*Angiotensin I converting enzyme* (*ACE)*	I/D alleles (rs4343)	Involved in regulating blood pressure
*Angiotensinogen (AGT)*	M235T (rs699)	Involved in regulating blood pressure
	T174M (rs4762)	
	A-20C (rs5050)	
	A-6G (rs5051)	
*Apolipoprotein A-I (APOA1)*	G-75A (rs670)	Component of HDL particles
	84 T/C (rs5070)	
*Apolipoprotein A-IV (APOA4*)	Asn147Ser (rs5104)	Activates lecithin-cholesterol acyltransferase (LCAT)
*Apolipoprotein A-V (APOA5)*	T-1131C (rs662799)	Determinant of plasma TG level
	−3 A/G (rs651821)	
	S19W (rs3135506)	
	Gly185Cys (rs2075291)	
*Apolipoprotein B (APOB*)	XbaI (rs693)	Component of LDL particles
*Apolipoprotein C-III (APOC3)*	SstI (rs5128)	Major component of TG-rich lipoproteins
	T-455C (rs2854116)	
*Apolipoprotein E (APOE)*	Cys112Arg (rs429358)	Acts as a ligand receptor in the process of catabolism of lipoproteins
	Arg158Cys (rs7412)	
	G-219T (rs405509)	
*ATP-binding cassette, sub-family A (ABC1), member 1 (ABCA1*)	Arg219Lys (rs2230806)	Transports cholesterol and phospholipids through the cell membrane to outside the cell
*Cholesteryl ester transfer protein, plasmatique (CEPT)*	TaqIB (rs708272)	Transfers cholesterol between lipoproteins
	C-629A (rs1800775)	
	C-4502T (rs183130)	
	G-971A (rs4783961)	
	Ile405Val (rs5882)	
*Cytochrome P450, family 1, subfamily A, polypeptide 1 (CYP1A1)*	Msp1 (rs4646903)	Catalyses reactions involved in drug metabolism and synthesis of cholesterol, steroids and other lipids
	A4889G (rs1048943)	
*Fat mass and obesity associated (FTO)*	rs9939609	Linked to obesity and diabetes
*Glucokinase (hexokinase 4) regulator (GCKR)*	rs780094	Inhibitor and glucokinase
*Insulin induced gene 2 (INSIG2*)	rs7566605	Connected to the metabolism of lipids, cholesterol, and obesity
*Lipoprotein lipase (LPL*)	HindIII (rs320)	Hydrolyses TG-rich lipoproteins
	Ser447Ter (rs328)	
*Lipase (hepatic) (LIPC (HL))*	C-514T (rs1800588)	Hydrolyses TGs and phospholipids of plasma lipoproteins
	G-250A (rs2070895)	
*Methylenetetrahydrofolate reductase (NAD(P)H) (MTHFR*)	C677T (rs1801133)	Catalyzes the conversion of a co-substrate for homocysteine
*Paraoxonase 1 (PON1)*	L55M (rs854560)	Reduces LDL-oxidation
	Gln192Arg (rs662)	
	C-107T (rs705379)	
*Peroxisome proliferator-activated receptor gamma 2 (PPARG2)*	Pro12Ala (rs1801282)	Regulator of adipocyte differentiation
	−681 C/G (rs10865710)	
*Transcription factor 7-like 2 (TCF7L2)*	C47833T (rs7903146)	Transcription factor

### Statistical analysis

Hardy-Weinberg equilibrium (HWE) was tested with the Allele Procedure in SAS, version 9.2 (SAS Institute Inc, Cary, NC). Distribution of alleles between the Inuit and Caucasian populations has previously been reported [7]. After completion of the recruitment, a group of 501 participants was calculated to provide an 80% probability and 5% significance level of detecting an anticipated difference of 0.10 mmol/L for TG concentrations (primary outcome) with a genetic variation that occurs in a relatively low frequency (>10%) of the population.

Data are shown as mean ± standard error of mean (S.E.M). Further, variables were checked for normality. Plasma TG and ratio TC/ HDL-C were transformed by natural logarithm to normalize their distribution. A statistical model was used to evaluate the effect of the polymorphism, RBC percentage of total *n*-3 PUFA as a continuous variable, and the polymorphism by RBCs percentage of total *n*-3 PUFA interaction effect, adjusted for the effects of age, sex, BMI, and total energy intake (kcal) on each of the lipid variables. Then, the absolute values of the regression beta- (β) coefficient were derived to estimate the phenotypic difference contributed by each of the genotype by RBC percentage of total *n*-3 PUFA interaction effect. In addition, to test for the additive effects of multiple polymorphisms, a “risk score” was calculated based on the number of risk genotypes an individual carried from all the significant polymorphism by RBCs percentage of total *n*-3 PUFA interactions for each lipid parameter. A statistical model was used to evaluate the effect of the “risk score”, RBCs percentage of total *n*-3 PUFA as a continuous variable, and the risk score by RBCs percentage of total *n*-3 PUFA interaction effect, adjusted for the effects of age, sex, BMI and energy intake (kcal) on each of the lipid variables. Again, the absolute value of the regression β- coefficient was calculated to estimate the phenotypic difference contributed by the number of risk genotypes and percentage of total *n*-3 PUFA in RBCs interaction effects. When significant differences were found, a comparison pairwise was performed in a global analysis (Tukey tests; significance *P* ≤ 0.05). Statistical analyses were performed with SAS statistical software, version 9.2 (SAS Institute Inc, Cary, NC).

## Results

### Phenotypic characteristics

In summary, 677 households were contacted of which 521 agreed to participate including 1056 individuals who signed a consent form and 917 who agreed to the collection of blood samples. In addition, 769 participants completed the FFQ. For the additional genetic component, 658 individuals from the original individuals gave consent. Consequently, the statistical analyses were done for all individuals where plasma lipid concentrations, percentage of total *n*-3 PUFA in RBCs, energy intake and genotypes were available. Therefore, 553 participants were included. The baseline characteristics, plasma lipids levels, percentage of total *n*-3 PUFAs in RBCs, and daily energy intake are described in Table 2. Briefly, higher RBC percentages of *n*-3 PUFA in the Inuit population were associated to lower TG concentrations (*P* = 0.0119), higher TC (*P* = 0.0001) including higher HDL-C (*P* < 0.0001) and higher LDL-C concentrations (*P* = 0.0146) without changing the TC/HDL ratio and plasma apoB100 levels when adjusted for age, sex, BMI and energy intake.

**Table 2:** Baseline characteristics of the study subjects

	**Men (n = 251)**	**Women (n = 302)**
**Anthropometrics**		
Age	37.1 ± 0.9	37.3 ± 0.8
Weight (kg)	74.5 ± 1.0	65.9 ± 0.9
Height (cm)	165.8 ± 0.4	154.0 ± 0.3
BMI (Kg/m^2^)	27 ± 0.3	28 ± 0.4
Hypercholesterolemia (n,% of total)	22 (9%)	21 (7%)
**Plasma Lipids**		
Total Cholesterol (mmol/L)	4.94 ± 0.06	5.04 ± 0.05
HDL- Cholesterol (mmol/L)	1.48 ± 0.02	1.79 ± 0.03
LDL- Cholesterol (mmol/L)	2.89 ± 0.06	2.74 ± 0.05
Total/HDL- Cholesterol ratio	3.52 ± 0.07	2.96 ± 0.05
Triglycerides (mmol/L)	1.25 ± 0.04	1.11 ± 0.04
ApoB100 (mmol/L)	0.97 ± 0.02	0.93 ± 0.01
ApoA1 (mmol/L)	1.62 ± 0.02	1.77 ± 0.02
***n*-3 PUFA in RBCs**		
Eicosapentaenoic acid (EPA) (%)	1.52 ± 0.08	1.82 ± 0.07
Docosapentaenoic acid (DPA) (%)	2.17 ± 0.03	2.21 ± 0.03
Docosahexaenoic acid (DHA) (%)	5.11 ± 0.12	5.80 ± 0.11
Total *n*-3 PUFAs (%)	9.14 ± 0.22	10.19 ± 0.20
**Daily Intake**		
Total Energy Intake (KJ)	10932 ± 352	9291 ± 301

Total percentage of n-3 PUFAs in RBCs was calculated from the sum of C18:3 (n-3), C18:4 (n-3), C20:4 (n-3), C20:5 (n-3) (EPA), C22:3 (n-3), C22:5 (n-3) (DPA), and C22:6 (n-3) (DHA).

### Genotypic characteristics

Genotype frequencies did not deviate from those predicted by the Hardy-Weinberg equilibrium except for the following 3 SNPs: *LPL* HindIII (rs320), *LPL* Ser447Ter (rs328) and *TCF7L2* C47833T (rs7903146) which were excluded from statistical analyses. The minor alleles of the following SNPs: *AGT* T174M (rs4762), *AGT* A-20C (rs5050), *APOA5* Gly185Cys (rs2075291), *APOA5* S19W (rs3135506), *APOE* Cys112Arg (rs429358), and *FTO* (rs9939609) had low frequencies (MAF < 0.10) in the Inuit population and were also excluded from analyses. In addition, allele frequencies were significantly different (*P* ≤ 0.05) in 18 SNPs in the Inuit population comparatively to the Caucasian population, as previously described [7].

### Gene- diet interactions for CVD risk factors

Statistical analyses revealed 8 SNPs by percentage of *n*-3 PUFA in RBCs interactions for plasma lipid concentrations (Table 3)*.* Briefly, subjects heterozygous for *CETP* C4502T (rs183130) or G-971A (rs4783961) had significantly lower plasma TG concentrations with higher *n*-3 PUFA percentage in RBCs compared to homozygotes for the minor allele. In addition, a more favorable plasma lipid profiles when higher percentages of *n*-3 PUFA were observed: lower plasma TC for T carriers of *CETP* C4502T (rs183130) or lower plasma TC/HDL ratio for *CETP* G-971A (rs4783961) for AG genotype. Participants homozygous for the T allele of *CETP* Ile405Val (rs5882) had lower plasma TC and T carriers of *CETP* Ile405Val (rs5882) had higher plasma HDL-C concentrations when higher percentage of *n*-3 PUFA in RBCs were observed. Secondly, for *APOA5,* G carriers of −3 A/G (rs651821) or C carriers of T-1131C (rs662799) exhibited higher plasma LDL-C concentrations, lower plasma HDL-C concentrations, a higher plasma TC/HDL ratio as well as higher apoB100 levels when they had a higher percentage of total *n*-3 PUFA in RBCs. Thirdly, carriers of the mutated allele of *AGT* M235T (rs699), compared to wild-type genotype, had decreased plasma TC and LDL-C concentrations when higher *n*-3 PUFA percentage were observed. Forth, G allele carriers of *APOA4* Asn147Ser (rs5104) had greater apoB100 concentrations with higher percentage of *n*-3 PUFA. Finally, subjects carrying the G allele of *APOC3* SstI (rs5128) had increased TC/ HDL ratio and apoB100 concentrations with higher percentage of *n*-3 PUFA. Additionally, a higher number of these above “risk alleles” in interaction with percentage of total n-3 PUFA were associated with significantly higher TG (*P* = 0.0069), TC (*P* = 0.0010), LDL-C (*P* = 0.0025) and apoB100 (*P* = 0.0044) concentrations as well as TC/HDL-C ratio (*P* = 0.0004) together with lower HDL-C (*P* = 0.0030) concentrations. Overall, polymorphisms in *CETP, AGT, APOA5, APOA4* and *APOC3* and the percentage of *n*-3 PUFA in RBCs are associated with plasma lipid concentrations in the Inuit population.

**Table 3:** Impact of the SNP, RBCs total n-3 PUFA and the interaction SNP * RBCs total n-3 PUFA on plasma lipids and lipoproteins levels

	**SNP**	**P-value for SNP**	**P-value for n-3 PUFA**	**Genotype/ Frequency**	**β** ± **SEM**	**P-Value for Interaction effect**
**Total cholesterol (TC)**	*AGT*	0.1453	0.4455	C/C (71%)	0^a^	0.0448
	M235T			C/T (26%)	0.0210 ± 0.0262^a^	
	rs699*			T/T (3%)	−0.1482 ± 0.0651^b^	
	*CETP*	0.0152	<0.0001	C/C (36%)	0^a^	0.0326
	C-4502T			C/T (49%)	−0.0632 ± 0.0241^b^	
	rs183130			T/T (15%)	−0.0421 ± 0.0343^ab^	
	*CETP*	0.1115	0.0002	C/C (26%)	0^ab^	0.0334
	Ile405Val			C/T (47%)	0.0397 ± 0.0268^a^	
	rs5882*			T/T (26%)	−0.0290 ± 0.0307^b^	
**LDL-Cholesterol (LDL-C)**	*AGT*	0.1514	0.6339	C/C (71%)	0^a^	0.0405
	M235T			C/T (26%)	0.0299 ± 0.0238 ^a^	
	rs699*			T/T (3%)	−0.1228 ± 0.0592^b^	
	*APOA5*	0.012	0.0018	A/A (42%)	0^a^	0.006
	−3 A/G			G/A (45%)	0.0678 ± 0.0213^b^	
	rs651821			G/G (13%)	0.0474 ± 0.0332^ab^	
	*APOA5*	0.0137	0.0035	T/T (42%)	0^a^	0.0072
	T-1131C			T/C (44%)	0.0669 ± 0.0213^b^	
	rs662799			C/C (34%)	0.0427 ± 0.0339^ab^	
**HDL-Cholesterol (HDL-C)**	*APOA5*	0.126	0.0002	A/A (42%)	0^a^	0.0065
	−3 A/G			G/A (45%)	−0.0317 ± 0.0100^b^	
	rs651821			G/G (13%)	−0.0105 ± 0.0155^ab^	
	*APOA5*	0.01476	0.0002	T/T (42%)	0^a^	0.0072
	T-1131C			T/C (44%)	−0.0314 ± 0.0100^b^	
	rs662799			C/C (34%)	−0.0115 ± 0.0159^ab^	
	*CEPT*	0.2302	0.0017	C/C (26%)	0^a^	0.0271
	Ile405Val			C/T (47%)	0.0263 ± 0.0115^b^	
	rs5882*			T/T (26%)	0.0017 ± 0.0131^a^	
**Triglycerides (TG)**	*CETP*	0.0092	0.3779	C/C (36%)	0^ab^	0.0300
	C-4502T			T/C (49%)	−0.0095 ± 0.0051^a^	
	rs183130			T/T (15%)	0.0073 ± 0.0073^b^	
	*CETP*	0.0087	0.399	G/G (28%)	0^ab^	0.0032
	G-971A			A/G (50%)	−0.0106 ± 0.0057^a^	
	rs4783961			A/A (22%)	0.0087 ± 0.0068^b^	
**Ratio Total Cholesterol/ HDL-Cholesterol**	*APOC3*	0.2263	0.7503	C/C (33%)	0^a^	0.0137
	SstI			C/G (46%)	0.0081 ± 0.0030 ^b^	
	rs5128*			G/G (21%)	0.0085 ± 0.0039 ^b^	
	*APOA5*	0.0016	0.5195	A/A (42%)	0^a^	<0.0001
	−3 A/G			G/A (45%)	0.0128 ± 0.0028 ^b^	
	rs651821			G/G (13%)	0.0053 ± 0.0044^ab^	
	*APOA5*	0.0018	0.62	T/T (42%)	0^a^	<0.0001
	T-1131C			T/C (44%)	0.0126 ± 0.0028^b^	
	rs662799			C/C (14%)	0.0050 ± 0.0045^ab^	
	*CETP*	0.188	0.8473	G/G (28%)	0^ab^	0.0483
	G-971A			A/G (50%)	−0.0055 ± 0.0033^a^	
	rs4783961			A/A (22%)	0.0022 ± 0.0039^b^	
**ApoB100**	*APOC3*	0.0374	0.0399	C/C (33%)	0^a^	0.0073
	SstI			C/G (46%)	0.0179 ± 0.0058 ^b^	
	rs5128*			G/G (21%)	0.0145 ± 0.0076 ^ab^	
	*APOA4*	0.0277	0.0221	A/A (33%)	0^a^	0.0052
	Asn147Ser			A/G (45%)	0.0178 ± 0.0058^b^	
	rs5104			G/G (22%)	0.0177 ± 0.0073^b^	
	*APOA5*	0.0006	0.091	A/A (42%)	0^a^	0.0002
	−3 A/G			G/A (45%)	0.0229 ± 0.0055^b^	
	rs651821			G/G (13%)	0.0086 ± 0.0086^ab^	
	*APOA5*	0.0009	0.0516	T/T (42%)	0^a^	0.0002
	T-1131C			T/C (44%)	0.0227 ± 0.0055^b^	
	rs662799			C/C (34%)	0.0117 ± 0.0088^ab^	

β regression coefficient, P values are calculated with normalized values; β regression coefficient are derived from absolute values, ^a,b^ Means with different subscript letters for one SNP are significantly different after adjustment for Tukey test; * Genotype frequency is different between the Inuit and Caucasian populations; The model includes the SNP, n-3 PUFA in RBCs, and the interaction term with adjustment for the effects of age, sex, BMI, and daily energy intake.

## Discussion

Results suggest that genetic factors and the percentages of *n*-3 PUFA in RBCs influence CVD risk factors in the Inuit population. Specifically, results demonstrate that 8 SNPs in 5 candidate genes: *CETP* ((rs5882) (rs183130) and (rs4783961)), *AGT* (rs699), *APOA5* ((rs651821) and (rs662799)), *APOA4* Asn147Ser (rs5104), and *APOC3* (rs5128), are associated with plasma lipid concentrations in interaction with the percentages of *n*-3 PUFA in RBCs in the Inuit population.

Higher percentage of *n*-3 PUFA in the Inuit population were associated with lower TG levels, higher TC including HDL-C and LDL-C concentrations without affecting the TC/HDL ratio and plasma apoB100 levels. In the same way, a meta-analysis that combined 21 trials with consumption of fish oil, reported a decrease in plasma TG concentrations and an increase in HDL-C and LDL-C concentrations without changes in TC concentrations [18]. Thus, the beneficial effects of higher percentages of *n*-3 PUFA in RBCs on CVD risk factors in the Inuit population are similar to those previously reported; however, gene-nutrient interactions contribute the inter-individual variability observed in the plasma lipid levels in this population.

Heterozygotes for *CETP* C4502T (rs183130) or G-971A (rs4783961) had lower plasma TG concentrations with higher percentages of *n*-3 PUFA compared to the homozygotes for the minor allele. In addition, these heterozygotes for *CETP* C4502T or G-971A had lower plasma TC levels or TC/HDL ratio with higher percentages of *n*-3 PUFA compared to homozygotes for the rare allele, respectively. CETP plays a central role in HDL-C metabolism by shuttling cholesteryl esters from HDL particles to apoB- containing particles, partly in exchange for TG [19]. It is well-known that several genetic variants in the *CETP* gene are associated with altered plasma HDL-C concentrations, lipoprotein particle sizes, CETP plasma concentrations and activity, and perhaps the risk of coronary artery disease [19]. Earlier, Corella et al., [20] demonstrated no gene-environment interactions, including total fat, saturated fat and monounsaturated fat- for the *CETP* C4502T SNP on HDL-C concentrations. We have also demonstrated no interaction with total fat or saturated fat intake with this SNP on plasma lipid levels [7]; however, in the present study, the inclusion of *n*-3 PUFA in the diets of wild-type or heterozygotes for *CETP* would favor an improved cardiovascular risk profile compare to carriers of the minor allele.

Further, carriers of the T allele for *CETP* Ile405Val (rs5882) decreased plasma TC and increased plasma HDL-C concentrations with higher percentages of *n*-3 PUFA. This functional SNP, corresponding to the substitution of a valine for an isoleucine caused by a C → T mutation, has been associated with lower CVD risk via lowering of CETP protein concentration and activity; consequently, increasing HDL-C levels and lipoprotein particle sizes [21]. Previously, Darabi et al., [22] demonstrated that subjects carrying the C allele of *CETP* Ile405Val had greater reduction in plasma HDL-C and apoAI concentrations than subjects carrying a T allele when a high-PUFA: saturated diet was replaced with a low- PUFA: saturated diet [22]. Together, these studies suggest that PUFA intake in individuals with *CETP* Ile405Val polymorphism contributes to the variability of plasma lipids concentrations.

Similarly, higher percentage of *n*-3 PUFA in carriers of the T allele of *AGT* M235T (rs699) were associated with lower plasma TC and LDL-C concentrations than carriers of the wild-type allele. The M235T polymorphism in the *AGT* gene has been associated to an increased CVD risk in the presence of hypercholesterolemia [23]. In a previous study with this Inuit population, we demonstrated that carriers of the minor allele of M235T *AGT* with high total and saturated fat intake exhibited higher plasma TC and LDL-C concentrations [7]. Taken together, these results suggest that carriers of the *AGT* M235T should consume a diet high in *n*-3 PUFA and low in saturated fat, to reduce their risk of CVD.

In contrast, G allele carriers of *APOA5* -3 A/G (rs651821) or C allele carriers of *APOA5* T-1131C (rs662799) had a more deteriorated lipid profile including higher plasma LDL-C and apoB100 concentrations, a higher TC/HDL ratio together with lower plasma HDL-C concentrations with higher percentage of *n*-3 PUFA in RBCs. APOA5 regulates TG metabolism; therefore, different SNPs in *APOA5* may be associated with TG concentrations and CVD risk factors [24]. Variations in this gene have been previously associated with plasma lipid levels via modulation of dietary intake. First, an interaction of the *APOA5* T-1131C polymorphism with energy intake and total fat was observed for plasma TG and TC concentrations in a population of Puerto Rican older adults [25]. Secondly, a study in the Mediterranean population reported a positive association between fat intake and obesity in homozygotes for the T allele of *APOA5* T-1131C, whereas in subjects carrying the *APOA5* T-1131C polymorphism, higher fat intakes were not associated with higher BMI and TG-rich lipoproteins [26]. Finally, Lai et al., [27] suggest that high PUFA-rich diets, specifically *n*-6 PUFAs, are related to a more atherogenic lipid profile in individuals of the Framingham Heart Study with the *APOA5* T-1131C polymorphism. Overall, results suggest that SNPs in *APOA5* are determinants of variation in lipid response to n-3 PUFA intake; however, the outcome of these gene-nutrient interactions needs to be investigated further.

Further, carriers of the G allele of *APOA4* Asn147Ser (rs5104) had higher apoB100 levels with higher percentages of *n*-3 PUFA in RBCs. It has been suggested that APOA4 plays an important role in the metabolism of TG-rich lipoproteins [28]. *APOA4* variants may alter protein and thus have been linked to baseline TG concentrations [28] and TG in response to fenofibrate treatment [29]. A study demonstrated that carriers of the minor allele of *APOA4* Asn147Ser had lower plasma apoA1 concentrations in a postprandial state after a high saturated meal [24]. Together, results demonstrate that carriers of the minor allele of *APOA4* Asn147Ser may be more sensitive to changes in dietary fat.

Similarly, carriers of the *APOC3* G minor allele of SstI (rs5128) had increased TC/HDL ratio and plasma apoB100 concentrations with higher percentages of *n*-3 PUFA. APOC3 increases plasma TG concentrations by inhibiting the lipoprotein lipase activity and by altering the Apo-E mediated uptake of TGs. The G- allele has been associated with elevated plasma TC, TG, and apoC-III concentrations [30]. Lopez-Miranda et al., [31] demonstrated an increase in plasma LDL-C concentrations in the CC subjects after consumption of a diet high in monounsaturated fatty acids whereas a decrease was observed in the CG subjects. Taken as a whole, results demonstrate that carriers of the *APOC3* G allele of SstI may benefit more from a high monounsaturated diet compared to an *n*-3 PUFA diet.

Since the effect size of each SNP was small even if the interaction was statistically significant, we demonstrated an additive effect of these SNPs in interaction with percentage of *n*-3 PUFA for all CVD risk factors. Thus, this demonstrates that it is possible to develop models with advantageous and disadvantageous alleles together with dietary intake data and predict the risk of a deteriorated lipid profile in a specific population. However, the major limitation for the study is the limited number of SNPs chosen based on previous research findings. If such models would be developed, large studies using numerous genetic variations should be included to be able to assess the greatest variability.

## Conclusions

Overall, these results suggest that the percentages of *n*-3 PUFA in RBCs, as an indicator of *n*-3 PUFA intake and genetic variations in *CETP*, *AGT*, *APOA5*, *APOA4* and *APOC3*, in the Inuit population are associated with CVD risk factors. In general, *n*-3 PUFA consumption has favorable effects on lipid related CVD risk factors; however, some genetic subgroups may benefit to greater extent from a higher intake of *n*-3 PUFA for lipid related CVD risk factors.

## Abbreviations

FAs: Fatty acids; CVD: Cardiovascular disease; RBCs: Red blood cells; PUFA: Polyunsaturated fatty acids; TG: Triacylglycerol; TC: Total cholesterol; LDL-C: Low-density lipoprotein cholesterol; HDL-C: High-density lipoprotein cholesterol; IHD: Ischemic heart disease; apo: Apolipoprotein; EPA: Eicosapentaenoic acid; DHA: Docosahexaenoic acid; DPA: Docosapentaenoic acid; ACE: Angiotensin I converting enzyme; AGT: Angiotensinogen; APOA1: Apolipoprotein A-I; APOA4: Apolipoprotein A-IV; APOA5: Apolipoprotein A-V; APOB: Apolipoprotein B; APOC3: Apolipoprotein C-III; APOE: Apolipoprotein E; ABC1: ATP-binding cassette, sub-family A; ABC1A: Member 1; CETP: Cholesteryl ester transfer protein; CYP1A1: Cytochrome P450, family 1, subfamily A, polypeptide 1; FTO: Fat mass and obesity associated; GCKR: Glucokinase (hexokinase 4) regulator; INSIG2: Insulin induced gene 2; LPL: Lipoprotein lipase; HL or LIPC: Hepatic lipase; NAD(P)H MTHFR: Methylenetetrahydrofolate reductase; PON1: Paraoxonase 1; PPARG2: Peroxisome proliferator-activated receptor gamma 2; TCF7L2: Transcription factor 7-like 2; HWE: Hardy-Weinberg equilibrium.

## Competing interests

The authors declare that they have no competing interests.

## Authors’ contributions

IR performed statistical analysis, interpreted data and wrote the paper; CO performed statistical analysis; VB, ADL, BA, FP, YG, and MLCD were responsible for data collection and analysis including genotyping, nutrient intakes and lipid parameters in the original study; ED, RAH and MCV were responsible for the study design; all authors contributed to the critical revision of the article; IR and MCV have primary responsibility for final content. All authors read and approved the final manuscript.
